# Whole-Genome Sequences of 14 Strains of *Bradyrhizobium canariense* and 1 Strain of *Bradyrhizobium japonicum* Isolated from *Lupinus* spp. in Algeria

**DOI:** 10.1128/genomeA.00676-17

**Published:** 2017-07-20

**Authors:** Djamel Chekireb, Julien Crovadore, Andreas Brachmann, Romain Chablais, Bastien Cochard, François Lefort

**Affiliations:** a Laboratory of Microbiology, Department of Biochemistry, Faculty of Sciences, University Badji Mokhtar Annaba, Annaba, Algeria; b Plants and Pathogens Group, Research Institute Land Nature and Environment, hepia, HES-SO University of Applied Sciences and Arts Western Switzerland, Jussy, Geneva, Switzerland; c Faculty of Biology, Genetics, Ludwig-Maximilians-University Munich, Planegg-Martinsried, Germany

## Abstract

We report here the whole-genome sequences of 14 strains of* Bradyrhizobium canariense*, isolated from root nodules of *Lupinus microanthus* and *Lupinus angustifolius*, and 1 strain of *Bradyrhizobium japonicum* isolated from root nodules from *Lupinus angustifolius* in Algeria. These sequences add to the known diversity of this agronomically important genus.

## GENOME ANNOUNCEMENT

*Bradyrhizobium canariense* (1), a sister species of* Bradyrhizobium japonicum* (2), was described in 2005 as a bacterium that nodulates legumes of the tribes Genisteae and Loteae, but not *Glycine*, while *Bradyrhizobium japonicum* forms root nodules in important crops, such as soybean (*Glycine max*), mungbean (*Vigna radiata*), cowpea (*Vigna unguiculata*), and siratro (*Macroptilium atropurpureum*) (3, 4). Bacteria of both species are aerobic Gram-negative motile rods which do not form spores, grow slowly, and are highly acid tolerant (1, 2, 5). Both species produce exopolysaccharides, and the culture phenotypes are diverse among the strains. They are found as free-living organisms in soils or as plant symbionts in root nodules. Initially described from root nodules from legumes of the Canary Islands (1), *Bradyrhizobium canariense* has since been found at many locations, such as Poland (6, 7), Italy and central Europe (7), Greece (8), and Morocco (9). While diverse strains of *Bradyrhizobium japonicum* are used as seed inoculants in *Glycine max* cultivation, the interest in using strains of* Bradyrhizobium canariense* as seed inoculants for cultures of *Lupinus* spp. and *Ornithopus compressus* (serradella) is rather recent (8). The 15 strains described here were isolated from root nodules of *Lupinus angustifolius* and *Lupinus micranthus* (Papilionoideae: Genisteae), collected at 2 sites in the National Park El-Kala (El-Tarf, Algeria). For whole-genome sequencing, DNA libraries were generated with a Nextera XT kit (Illumina, USA). Sequencing was performed on a MiSeq sequencer (Illumina) in three different runs generating 2 × 250-bp paired-end reads (version 2 chemistry) and 2 × 250-bp and 2 × 300-bp paired-end reads (version 3 chemistry). Quality control of the reads was assessed with FastQC (http://www.bioinformatics.babraham.ac.uk/projects/fastqc). Genome assemblies were computed with SPAdes genome assembler 3.10 (10) and resulted in between 163 contigs (UBMA510) and 235 contigs (UBMA181) per genome, which were arranged with BioEdit (11) and analyzed with QUAST (12). For *Bradyrhizobium canariense* strains, the total genome lengths ranged between 8,220,547 bp (UBMA122) and 8,379,024 bp (UBMAN05), with G+C contents from 62.94% (UBMA052, UBMA183, and UBMA192) to 63.06% (UBMA122), and *Bradyrhizobium japonicum* UBMA197 had a larger genome, at 10,442,239 bp, with 63.3% G+C content. Genome coverage varied from 46-fold (UBMA510) to 255-fold (UBMA060). PlasmidFinder (13) and PlasmidSPAdes (14) detected no plasmids in these strains. RAST 2.0 (15, 16) identified between 6,304 (UBMAN05) and 7,974 (UBMA122) coding sequences for *Bradyrhizobium canariense* sequences and 10,125 coding sequences for *Bradyrhizobium japonicum* UBMA197. No photosystems, complete transposons or phages, or toxin genes were found in any of the strains. All strains had a nitrogenase capacity through a regulatory *nifA* gene and between 11 and 25 additional *nif* genes grouped in a genomic island. All strains were able to nodulate due to the presence of 9 to 12 *nod* genes typical of the *Bradyrhizobium* genus. The genomes also contained between 134 and 150 genes involved in the degradation of aromatic compounds. Finally, all strains were equipped with protein secretion systems of types II, IV, and VI, except for strain N05, which was almost deprived of such genes. The 15 presented genome sequences add to the knowledge of these species, which are considered potent inoculants for cultivation of certain leguminous plants (8).

### Accession number(s).

All genome sequences have been deposited at GenBank under the accession numbers reported in Table 1.

**TABLE 1:** Nucleotide sequence accession numbers

Species	Strain name	Genome accession no.	Contig accession no.
*B. canariense*	UBMA050	NAEX00000000	NAEX01000001 to NAEX01000188
*B. canariense*	UBMA051	NAEY00000000	NAEY01000001 to NAEY01000173
*B. canariense*	UBMA052	NAEZ00000000	NAEZ01000001 to NAEZ01000182
*B. canariense*	UBMA060	NAFA00000000	NAFA01000001 to NAFA01000204
*B. canariense*	UBMA061	NAFB00000000	NAFB01000001 to NAFB01000196
*B. canariense*	UBMA122	NAFC00000000	NAFC01000001 to NAFC01000177
*B. canariense*	UBMA171	NAFD00000000	NAFD01000001 to NAFD01000195
*B. canariense*	UBMA181	NAFE00000000	NAFE01000001 to NAFE01000227
*Bradyrhizobium* sp.	UBMA182	NAFF00000000	NAFF01000001 to NAFF01000114
*B. canariense*	UBMA183	NAFG00000000	NAFG01000001 to NAFG01000183
*B. canariense*	UBMA192	NAFH00000000	NAFH01000001 to NAFH01000180
*B. canariense*	UBMA195	NAFI00000000	NAFI01000001 to NAFI01000190
*B. japonicum*	UBMA197	NAFL00000000	NAFL01000001 to NAFL01000287
*B. canariense*	UBMA510	NAFJ00000000	NAFJ01000001 to NAFJ01000159
*B. canariense*	UBMAN05	NAFK00000000	NAFK01000001 to NAFK01000178
